# Post-intubation tracheal stenosis after severe COVID-19 infection: A report of two cases

**DOI:** 10.1016/j.amsu.2021.102468

**Published:** 2021-06-09

**Authors:** Ahmad Alturk, Albaraa Bara, Bassam Darwish

**Affiliations:** a Faculty of Medicine, Damascus University, Damascus, Syrian Arab Republic; b Department of Thoracic Surgery, Al-Mouassat University Hospital, Damascus, Syrian Arab Republic

**Keywords:** COVID-19, PILS, Stenosis, Resection, Case report

## Abstract

**Introduction and importance:**

Coronavirus disease 2019 (COVID-19) is a pandemic disease that spread rapidly throughout the world and became a major public health concern. Approximately 5–12% of COVID-19 patients require admission to the intensive-care unit (ICU), where they often require oxygen therapy and prolonged intubation. Post-intubation laryngotracheal stenosis (PILS) is a complication that occurs in 10–22% of non-COVID-19 patients after prolonged intubation, while the rate of COVID-19 related PILS remains unknown. Additionally, there is still no consensus in the literature regarding the management modalities for PILS following COVID-19.

**Case presentation:**

Here we report two cases of tracheal stenosis after prolonged intubation due to severe COVID-19 infection. The first patient was admitted to the ICU and intubated for 21 days; 3 months after discharge, he developed a 3 cm long tracheal stenosis that narrowed 70% of the lumen. The second patient was intubated for 2 months and, 4 months after discharge, developed a 2.5 cm long tracheal stenosis that narrowed 80% of the lumen.

**Clinical discussion:**

In both cases, the diagnosis was confirmed by CT scan and Rigid bronchoscopy; then, they were managed successfully with tracheal resection and reconstruction by end-to-end anastomosis.

**Conclusion:**

In conclusion, we would like to highlight the importance of suspecting PILS in recovered COVID-19 patients re-presenting with breathing difficulties following weaning from mechanical ventilation; therefore, careful follow-up in such patients is required. Moreover, we would like to point out that the management of tracheal stenosis after COVID-19 appears to be similar to that of tracheal stenosis in general.

## Highlights

• Tracheal stenosis (TS) is a rare complication that follows prolonged intubation.
• Prolonged intubation causes tracheal wall damage, which leads to TS.
• Suspect TS in recovered ICU-admitted COVID-19 patient re-presenting with dyspnea.
• Stenosis length, severity, and location are essential in the management plan.
• Consider non-surgical procedures in patients with “thin and young” stenoses.

## 1 Introduction

Coronavirus disease 2019 (COVID-19) is a pandemic infectious disease caused by a coronavirus called severe acute respiratory syndrome coronavirus 2 (SARS-CoV-2). The disease has spread rapidly across every country and has become a major public health concern [1]. Most COVID-19 patients present with minor symptoms like fever, cough, fatigue, headache, new loss of taste or smell, sore throat, nausea, and gastrointestinal symptoms [2]. In a low number of patients, the disease may take a severe form with serious complications, including interstitial pneumonia, acute respiratory failure, acute respiratory distress syndrome (ARDS), multiple organ failure, and death [3].

Only 5–12% of COVID-19 patients require admission to the intensive-care unit (ICU); such patients often require prolonged mechanical ventilation with high positive end-expiratory pressure (PEEP) through an endotracheal tube [2].

While tracheal intubation is a life-saving procedure, it may cause mucosal damage and tracheal wall necrosis, which leads to laryngotracheal stenosis [4]. The rate of post-intubation laryngotracheal stenosis (PILS) in non-COVID-19 patients is 10–22% [3]. Although the rate of COVID-19 related PILS remains unknown, it is thought that this complication is more common in COVID-19 patients after prolonged tracheal intubation [3]. On top of that, the choice of the modality of management of these patients is still undetermined.

In this paper, we report two cases of patients with post-intubation tracheal stenosis diagnosed during their recovery phase of COVID-19 and treated by surgical resection and reconstruction by end-to-end anastomosis. This work has been reported in line with the SCARE criteria [5].

## 2 Case reports

### 2.1 Case 1

A 35-year-old man presented to our hospital complaining of dyspnea with stridor that started a month before and exacerbated during the last week. The patient was a non-smoker and had no significant surgical or family history. 4 months before, he was admitted to the intensive care unit (ICU) and intubated for 21 days due to acute respiratory failure that occurred as a complication of COVID-19. On admission, his peripheral oxygen saturation (SpO_2_) was 96% on room air. CT scan showed signs of tracheal stenosis. He underwent 3 attempts of tracheal dilatation without a satisfactory outcome. Rigid bronchoscopy revealed a 3 cm long tracheal stenosis that narrowed 70% of the lumen; the stenosis was 5 mm below the cricoid cartilage. The stenosis was managed successfully with tracheal resection and reconstruction. The postoperative period was uneventful, and the patient was reassessed after 2 months: his general condition was good without complaints, and bronchoscopy revealed a non-narrowing anastomotic line.

### 2.2 Case 2

**Fig. 1:**
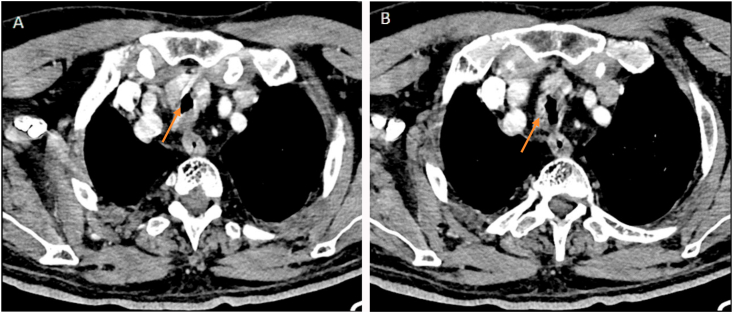
CT scan findings for Case 2. A significant tracheal stenosis after 2 months of intubation due to SARS-CoV-2 infection (A,B arrows).

A 62-year-old man presented to our hospital complaining of dyspnea and wheezing that started 2 months before and exacerbated during the last 10 days. The patient was a moderate smoker with a history of poorly controlled hypertension diagnosed 23 years ago. 6 months before, he was diagnosed with COVID-19 and admitted to the ICU due to the rapid deterioration of his respiratory condition. He was put on a mechanical ventilator for 2 months. On admission, his SpO_2_ was 95% on room air. CT scan showed signs of tracheal stenosis (Fig. 1). Rigid bronchoscopy revealed a 2.5 cm long tracheal stenosis that narrowed 80% of the lumen; the stenosis was 2 cm below the cricoid cartilage. We dilated the stenosis as a temporary solution, then we performed tracheal resection and reconstruction. The patient made a full recovery, and the postoperative period was uneventful.

## 3 Discussion

PILS is a rare condition with an estimated incidence of 4.9 cases per million per year in the general population [6]. PILS becomes symptomatic (1–2% of cases) when the stenosis includes >60% of the tracheal lumen. Previous reports showed that the time of onset of PILS varied from 28 days to 6 months after extubation [3]. The most common cause of PILS is iatrogenic [7], specifically in prolonged intubations, endotracheal tube cuff hyperinflation, and the usage of large tubes. Other risk factors include peritube infections, advanced age, female and estrogen effect, smoking, obesity, and diabetes.

There are still no data about the occurrence of PILS in recovered COVID-19 patients. However, it is thought that those patients could be at increased risk of developing PILS; perhaps most notably due to their hyperinflammatory state, which causes altered fibrosis, but also because those patients are subjected to prolonged intubations and usually already have comorbidities, such as diabetes and hypertension [7].

Early tracheostomy is recommended for patients who require prolonged tracheal intubation [8], which is the case of most COVID-19 patients admitted to the ICU [2]. However, the global trend for COVID-19 patients admitted to ICU is to postpone tracheostomy until the patient no longer needs mechanical ventilation and is cleared of the virus; this delay is mainly due to the high risk of cross-infection of healthcare professionals [6]. Early reports state that COVID-19 patients remained intubated from one to two weeks or longer [3]. In our case, one patient underwent 3 weeks and the other 8 weeks of mechanical ventilation.

Although Mr. Francesco Mattioli et al. [9] suggested managing tracheal stenosis that occurs after COVID-19 endoscopically with balloon dilation and local corticosteroid injection, which maintains an adequate airway that enables breathing for an acceptable period, he mentioned that this procedure could be temporary, and other procedures may be needed later; probably tracheal resection and reconstruction, as we did to our patients, who were both suitable for surgery. In another paper [7], Mr. Carmine Fernando Gervasio et al. reported managing two cases of tracheal stenosis following COVID-19: one with intravenous corticosteroids, while the other with tracheal resection. Nevertheless, we concur with Mr. Mattioli that dilation should be performed in patients with “thin and young” stenosis or when surgery is not feasible because the patient has comorbidities, which is the case of the majority of COVID-19 patients that needed prolonged intubation. Consequently, dilation and other procedures that sustain the airway patency, such as tracheal stent placement, are temporary procedures performed when the patient cannot withstand surgery or when the tracheal stenosis is not suitable for resection and reconstruction by end-to-end anastomosis (in the case of long stenosis or tracheal mucosa infection).

As an approach for tracheal stenosis, the location, length, and severity of the stenosis are essential factors in the management plan. If the stenosis is suitable for resection and reconstruction by end-to-end anastomosis and the patient can withstand the operation, surgery is recommended; otherwise, other therapeutic procedures are performed. Thus, the management strategies for tracheal stenosis after COVID-19 are similar to those for tracheal stenosis in general.

## 4 Conclusion

We presented two cases of post-intubation tracheal stenosis that occurred after COVID-19. It should highlight the importance of suspecting tracheal stenosis in recovered COVID-19 patients re-presenting with breathing difficulties following weaning from mechanical ventilation; therefore, careful follow-up in such patients is required. Moreover, we would like to point out that the management of tracheal stenosis after COVID-19 appears to be similar to that of tracheal stenosis in general.

## Informed consent

Written informed consent was obtained from our two patients for publication of this case report and accompanying images. A copy of the written consent is available for review by the Editor-in-Chief of this journal on request.

## Provenance and peer review

Not commissioned, externally peer-reviewed.

## Sources of funding

None.

## Ethical approval

None.

## Research registration

None.

## Author contribution

AB: reviewed the literature, wrote the abstract, and the introduction.

AA: reviewed the literature, wrote the case presentation, and the discussion.

BD: lead the surgical team, checked the spelling and grammar, revised the manuscript and helped in writing the discussion.

## Declaration of competing interest

All of the authors declared that they have no conflict of interest.
